# Prostate carcinoma metastatic to the skin as an extrammamary Paget’s disease

**DOI:** 10.1186/1746-1596-7-106

**Published:** 2012-08-18

**Authors:** Eugen Bogdan Petcu, Aldo Gonzalez-Serva, Robert G Wright, Mark Slevin, Klara Brinzaniuc

**Affiliations:** 1 Griffith University School of Medicine, Gold Coast Campus, Griffith University, Southport, QLD 4222, Australia; 2 Department of Pathology, Winchester Hospital, Winchester, MA, 01890, USA; 3 Department of Pathology, Gold Coast University Hospital, Southport, QLD, 4215, Australia; 4 Manchester Metropolitan University, Angiogenesis and Vascular Biology Group, Manchester, UK; 5 Department of Anatomy and Doctoral School, University of Medicine and Pharmacy Targu Mures, Targu Mures 540000, Romania

## Abstract

**Aim:**

The current paper describes a case of prostatic adenocarcinoma metastatic to the skin presenting as an extrammamary Paget's disease, a very rare and poorly characterised morphological entity. We report a case of prostatic carcinoma metastatic to skin showing a pattern of extramammary Paget's disease which has not been clearly illustrated in the literature Case presentation: A 63 year-old man with prostatic adenocarcinoma developed cutaneous metastases after 16 years. The inguinal metastases were sessile and 'keratotic.' The tumour displayed solid, glandular areas as well as a polypoid region suggestive of extramammary Paget's disease were identified.

**Discussion and conclusions:**

We review the diagnostic criteria that have led to the correct histopathological diagnosis in this case. A differential diagnosis of the pagetoid spread in the skin and various forms of cutaneous metastases determined by a prostatic adenocarcinoma as well as the role of immunohistochemistry in establishing the prostatic origin are presented in the context of this case. Although, morphologically the cells presented in the skin deposits were not characteristic for adenocarcinoma of prostate, immunohistochemistry for PSA and PSAP suggested a prostatic origin.

**Virtual Slides:**

The virtual slide(s) for this article can be found here: http://www.diagnosticpathology.diagnomx.eu/vs/1395450057455276

## Introduction

Prostate adenocarcinoma is one of the most common cancers in Australia. The Melbourne Collaborative Cohort Study revealed that 8.4% of the subjects enrolled in the study developed over 15 years prostatic adenocarcinoma and more than 10% of these patients died [1]. While some long-term survivors develop an indolent disease without dissemination others develop early or even late metastases. Secondary deposits associated with prostatic adenocarcinoma are located with predilection in the bone system while skin metastasis represents an exceptional event [2]. Evaluation of these skin lesions should always include a thorough clinical examination, past history and histopathological evaluation. In some patients the history of prostatic adenocarcinoma is absent and in others the histology is not characteristic for a prostatic origin or the patients might have had cancers with various origins. In these cases, immunohistochemistry is an invaluable tool, the most commonly used markers being prostate-specific antigen (PSA) and prostate acid phosphatase (PSAP) [3]. Skin metastasis determined by a prostate adenocarcinoma may display a variety of patterns including the extrammamary Paget’s disease. However, at the present time, we do not understand the implication of this morphology for the aggressiveness of the primary cancer and the general outlook of the patient.

## Case report

**Figure 1:**
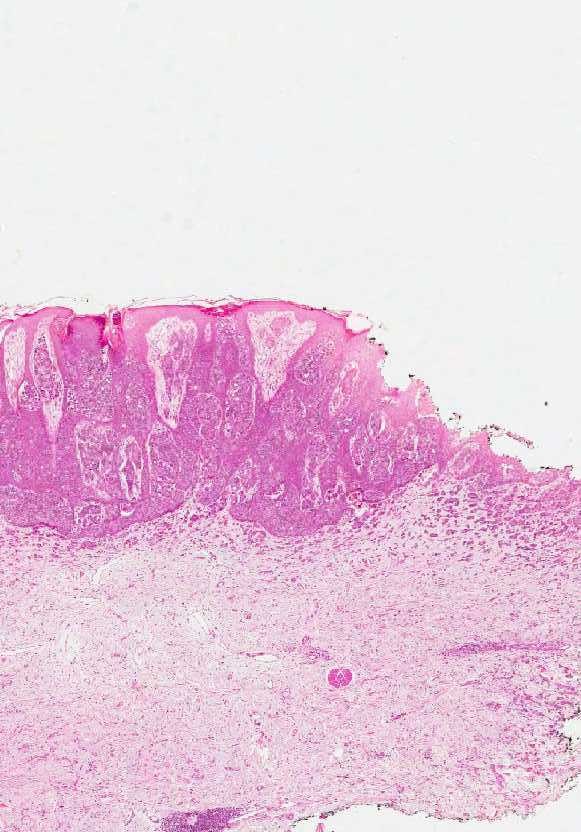
**Skin and subcutis showing hyperplastic epidermis with “clear cells” suggestive of EMPD.** The “shoulder” of the lesion reveals no epidermal involvement.

**Figure 2:**
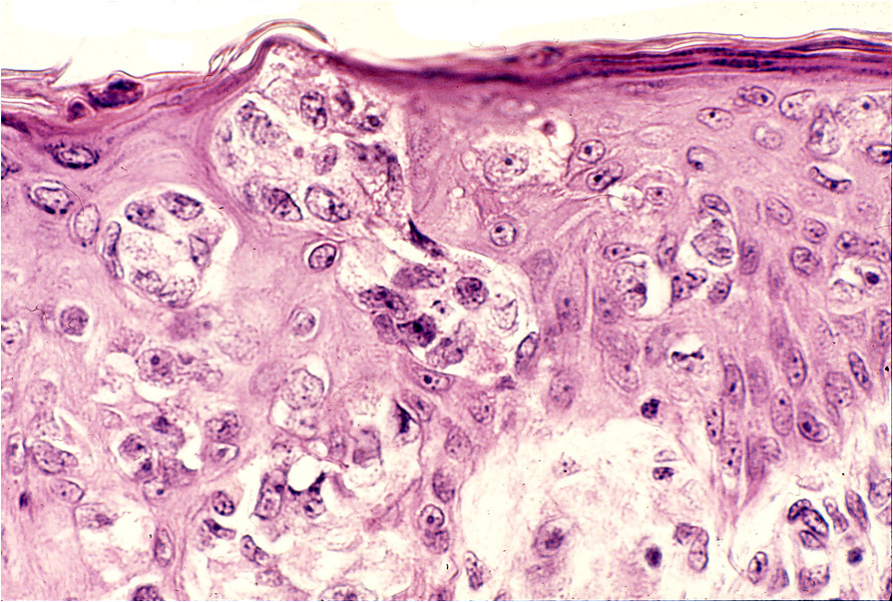
Hyperplastic epidermis showing massive infiltration by “clear cells” suggestive of EMPD.

Initially, a 63 year-old male was diagnosed with locally metastatic prostatic adenocarcinoma, moderately differentiated, Gleason score 3 + 3 = 6 (T3NxM0). However, no prostatectomy was performed after the initial clinical diagnosis. Subsequently, the patient elected to receive radiation therapy and long-term flutamide. A bone scan performed after eight years revealed no proliferative lesions. However, at 16 years after the initial diagnosis he was admitted to dermatology clinic with an eruption of multiple tan keratotic polypoidal lesions located on his scalp, abdomen and bilateral groin areas. Bilateral inguinal lymphadenopathy was also noted. Clinical and radiological evaluation revealed an irregularly enlarged prostate. The MRI showed abdominal lympadenopathy. Several atypical areas were detected in bone pelvis but a clear diagnosis of bone metastasis was not possible. However, no other masses were detected elsewhere. Immediately prior to his anatomo-pathological evaluation, the patient developed macroscopic haematuria, overflow urinary incontinence and renal failure with increased creatinine. The general status of the patient did not allow a prostatic biopsy and the patient was transferred for palliative care and expired after three weeks. However, during his hospital stay the PSA level increased from 24.3 ng/ml to 46.3 ng/ml. A skin biopsy of a fibroepithelial-like lesion located in the right lower abdominal quadrant (inguinal area) was performed a diagnosis of skin metastasis was made. Histopathological evaluation of this lesion revealed several patterns of metastatic prostatic adenocarcinoma. The dominant feature was represented by solid and glandular areas. In addition, large areas of hyperplastic epidermis revealed clear tumour cells suggestive of extramammary Paget’s disease (EMPD) (Figure 1; Figure 2).

## Discussion

The skin and subcutis represent the site for a large variety of epithelial stromal and lymphovascular tumours but also metastatic deposits are identified at this site. Thus metastases from most internal organs and breast have been described in the skin. The real incidence of skin metastasis determined by internal organs is difficult to be ascertained with precision but it seems to be between 2.8 and 5% [4,5]. In this context, prostate adenocarcinoma metastatic to the skin is an exceptional occurrence. Clinical research conducted in 4020 patients with cancer has revealed 207 cases of metastatic prostate carcinoma but none of the prostate cancer patients developed skin metastasis [6]. However, it is believed that when noted, skin metastases in patients with prostatic adenocarcinoma are indicative of a very poor outcome [7] and thorough clinico-pathological evaluation is mandatory. Rattanasirivilai et al. [8] mention fewer than 80 published reports of prostate adenocarcinoma metastatic to the skin between 1962 and 2009. However, a variety of morphological patterns have been described in the literature (Table 1)

**Table 1:** Cutaneous metastasis of prostatic adenocarcinoma: morphological patterns

• solid/poorly differentiated [9]
• glandular/ductal [10]
• infiltrative [11]
• mucinous with signet ring [12]
• teleangiectatic [13]
• lymphangitic [14]
• with epidermotropism [15]
• with small cell [16]

**Figure 3:**
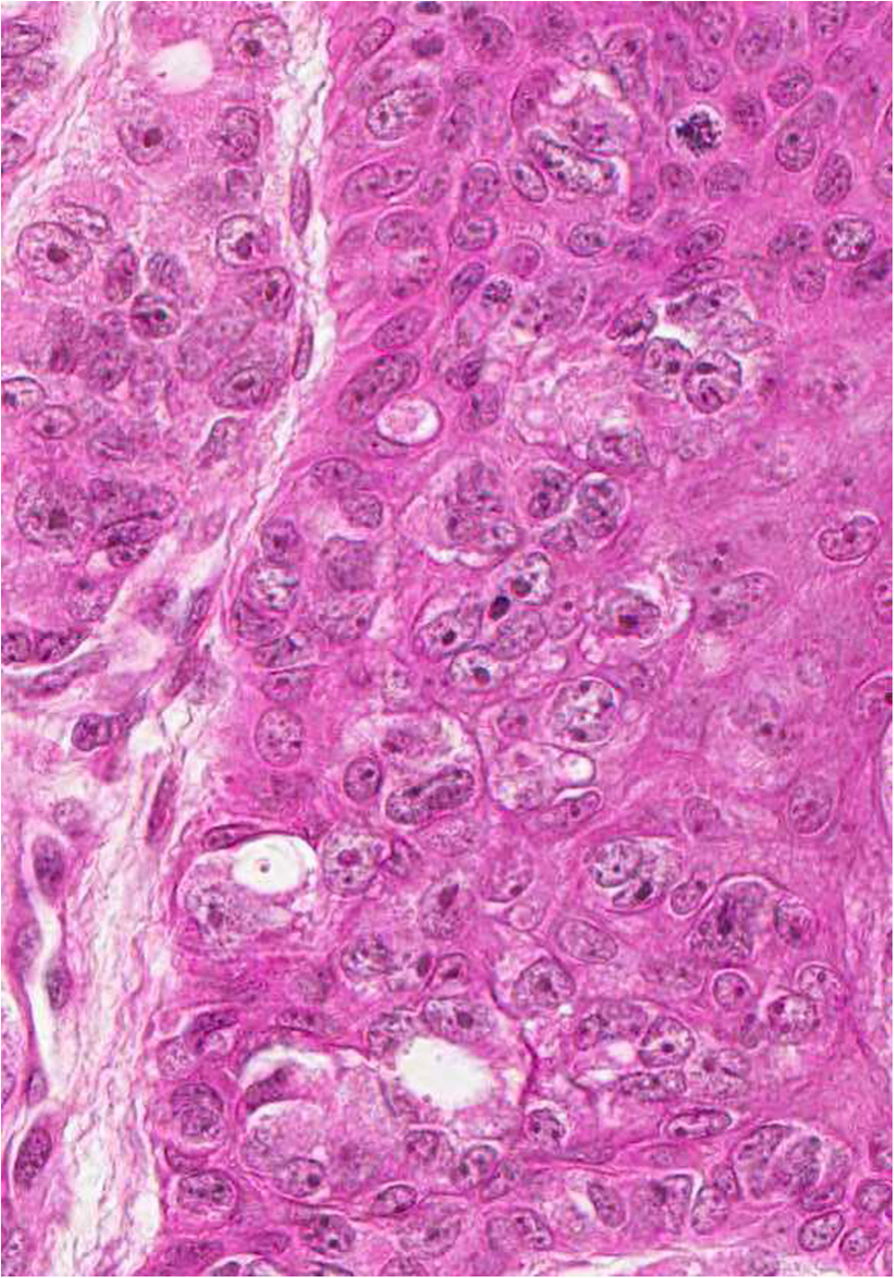
Malignant cells in epidermis: large, clear and clefted nuclei with atypical mitoses.

In our reported case, the background where the prostatic cells have lodged was represented by papulo-nodular skin with preservation of the adnexae (Figure 1). This obvious exophytic pattern was similar to that of fibroepithelial papillomas, melanocytic nevi or warts occurring in eyelids, neck, axilla or groin. Therefore, we conclude that a precise macroscopic differential diagnosis and a detailed personal history are of paramount importance for the initial clinical diagnosis and work-up. As mentioned, our patient presented with a mixed pattern of solid and glandular dissemination in the skin but the interesting aspect was the presence of neoplastic cells in the epidermis suggesting EMPD (Figure 3). These malignant cells present in the epidermis showed large clear and clefted nuclei with obvious nucleoli and atypical mitoses. Remarkably, some histological fields showed areas without evidence of EMPD especially towards the “shoulder” of the sections, which brings into question the evolution of the lesion from areas without EMPD to extensive epidermal metastasis regions.

Very importantly, this pattern of prostatic metastasis needs to be recognised since many other cancers present similarly and the differential diagnosis could be rather vast (Table 2).

**Table 2:** Pagetoid spread in the epidermis

**A. PRIMARY NEOPLASMS**
**Paget’s Disease (PD)**[17]
• Primary PD
• De novo in the areola [18]
**Melanocytic Tumors**[19]
• Malignant melanoma [20]
• Spitz’s nevus [21]
• Acral nevus [22]
**Epithelial Cutaneous Neoplasms**
• Squamous cell carcinoma (Bowen’s disease) [23]
• Extraocular sebaceous carcinoma of the shoulder [24] and left upper arm [25]
• Ocular sebaceous carcinoma (of Meibomian gland origin) in eyelids [26,27]
• Merkel cell carcinoma [28]
• Tricholemmal carcinoma [29]
• Porocarcinoma [30]
• Basal cell carcinoma [31]
**Benign Epidermal Conditions**
• Focal acantholytic dyskeratosis [32]
• Cutaneous hamartoma with pagetoid cells [33]
• Clear cell papulosis of the skin [34,35]
• Pagetoid dyskeratosis of the prepuce [36]
• Benign mucinous metaplasia of the penis (mucosal side of prepuce) [37]
• Mammary gland-related clear cells of normal nipples (Toker cells) [38,39]
**Lymphohematopoietic Conditions**
• Cutaneous T-cell lymphoma
• Pagetoid reticulosis
• Localized Woringer-Kolopp disease [40]
• and generalized Ketron-Goodman disease [41]
• Mycosis fungoides, common type
• Langerhans cell histiocytosis: self-healing [42], malignant [43], nodular [44]
• Leukemia: Monoblastic leukemia [45]
**B. METASTATIC NEOPLASMS**
Carcinomas and Malignant Melanoma

**Figure 4:**
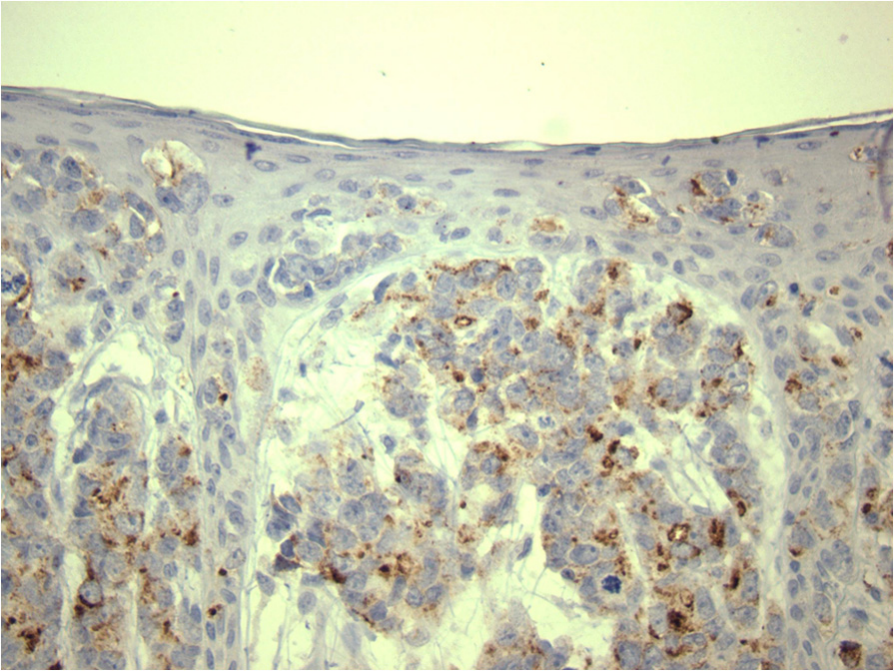
Malignant cells positive for PSA in both dermis and epidermis.

**Figure 5:**
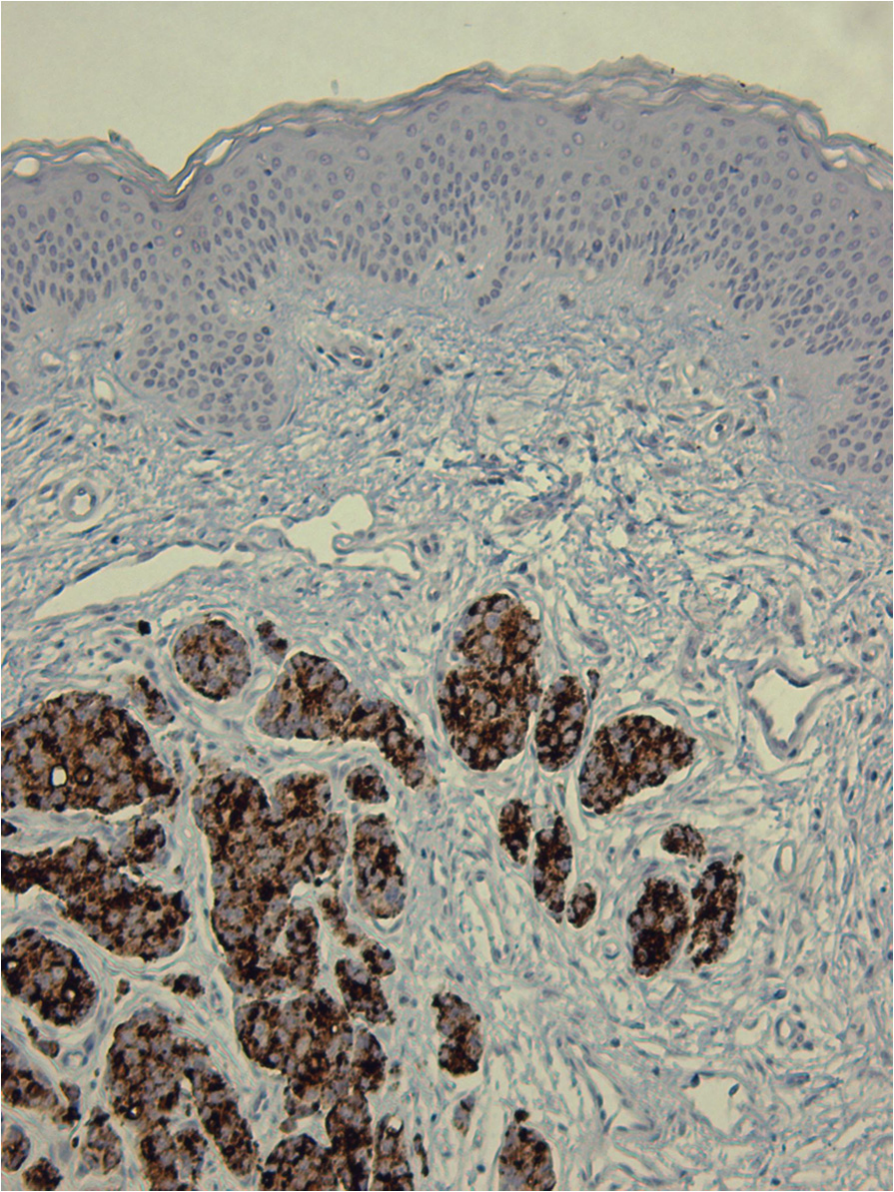
**Dermal clusters of malignant cells positive for PSA.** The above epidermis reveals no PSA positive neoplastic cells.

**Figure 6:**
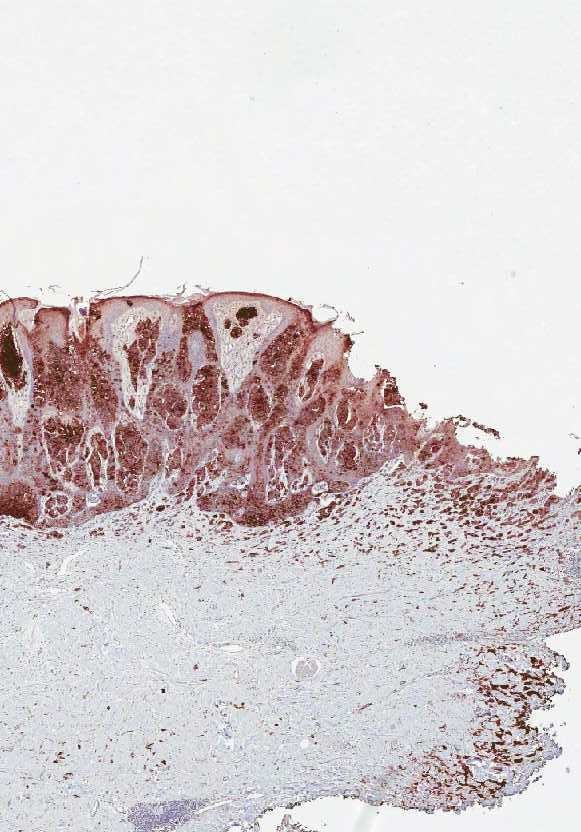
**Skin and subcutis showing hyperplastic epidermis with PSAP positive cells.** Dermal clusters of neoplastic cells show PSAP positivity as well.

**Figure 7:**
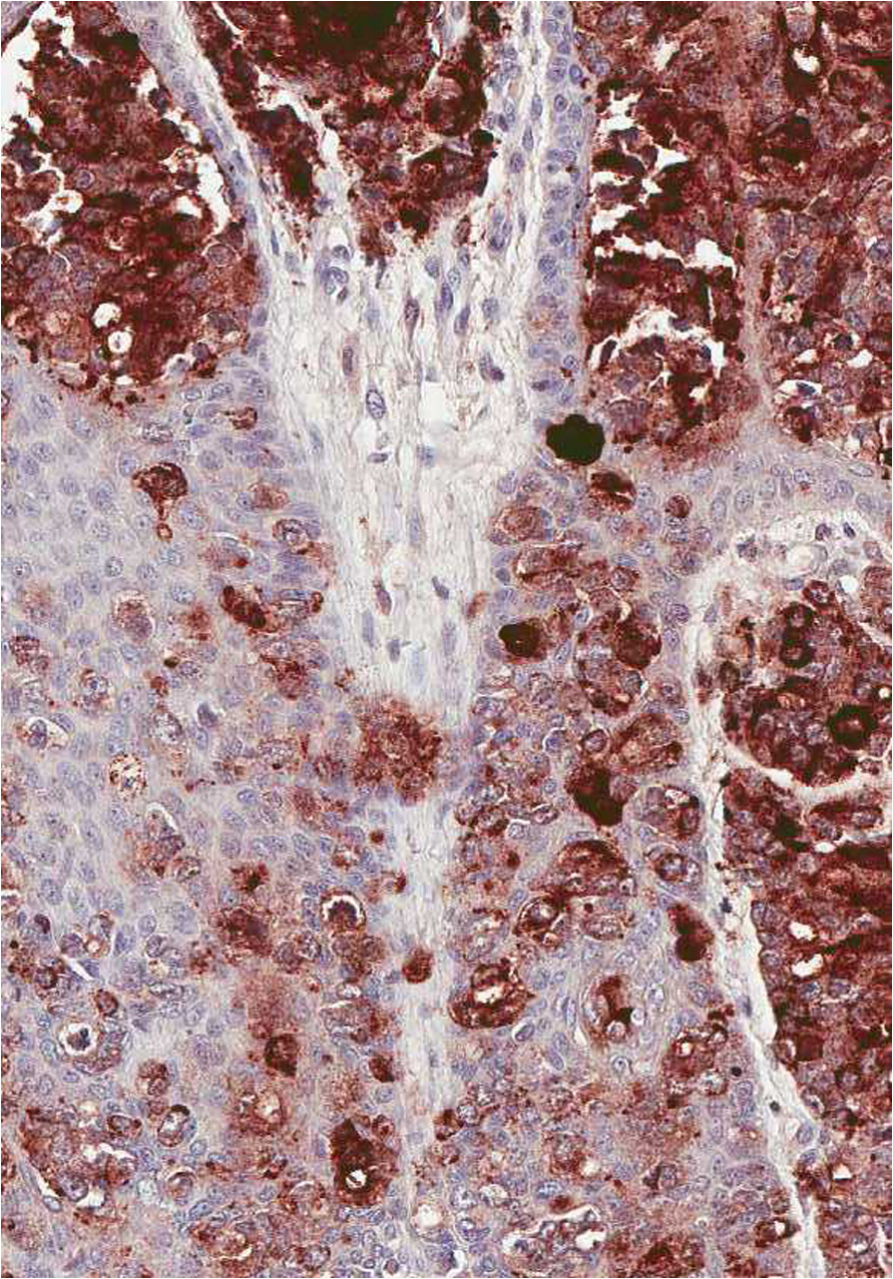
Dermal and epidermal PSAP positivity, high power.

The currently reported EMPD pattern in a site not prone to the development of a “primary” extrammamary Paget’s makes this lesion exceptional. However, the currently described lesion represents the dissemination from the initial prostatic adenocarcinoma rather than a “primary” EMPD. The first argument, upholding this would be the clinical history. It is likely that dormant neoplastic foci of prostatic adenocarcinoma have been reactivated. Subsequently, the patient has developed skin metastases. Reedy et al. [46] highlight the fact that although rare, the “primary”extrammamary Paget’s disease is usually seen as erythematous lesions in areas rich in apocrine glands such as axilla or perineum. Jones RE et al. [47] concluded in a study performed on fifty-five patients that in primary EMPD, the diagnostic criteria are represented by the Paget cells extending from the epidermis to the epithelial structures of adnexa, and the dermis. In our case, no lesional contiguity from the prostate to skin was present. Morphological changes described in this paper most likely represent the lymphovascular metastasis originating from the prostatic gland. One may argue that the aspect of the neoplastic cells seen in the epidermis is not that of usual prostatic adenocarcinoma. Most likely this represents a pleomorphic variant of prostatic cancer but since there is no direct link between the prostate and skin lesions, one may speculate that this patient had two primary lesions, in the skin and in the prostate. However, this is not the case and immunohistochemistry was of crucial importance in elucidating the diagnosis. Although, with the exception of very rare undifferentiated cases, the prostatic adenocarcinoma demonstrates positive staining for PSA. Contrary, some authors have suggested that skin metastases are negative [48] but in our case the PSA was intensely positive (Figure 4, Figure 5) for both dermal and epidermal metastatic cells. Interestingly, in examined tissue, the immunostaining pattern for PSA was displayed as clumps of brown staining unlike the finely granular staining classically described in prostatic cells. These findings raised again the question whether this is truly metastatic prostatic adenocarcinoma. It has been suggested that rarely several non-prostatic tumors such as salivary gland neoplasms, malignant melanoma, adenocarcinoma of paraurethral glands (Skene's), urothelial carcinoma may show PSA positivity [49]. However, on clinical, CT and MRI evaluation there was no evidence of any of the above mentioned tumours in our patient. Therefore, considering the past history and the current laboratory and histopathological information, the increased serum PSA level was more likely due to a prostatic adenocarcinoma metastatic to the skin. In addition, PSAP staining which is recommended if the PSA staining is not concludent was intensely positive and revealed neoplastic cells of prostatic origin in dermis and epidermis (Figure 6, Figure 7). The rest of immunohistochemistry markers including markers for neuroendocrine differentiation were non-contributory. The immunohistochemistry is of paramount importance in arriving at the correct diagnosis. Srinivasan et al. [50] have shown that double sequential staining for p63 and P501S (prostein) is very important to differentiate a prostatic carcinoma from an urothelial primary especially since some cases of urothelial carcinoma may present with increased PSA if they involve secondarily the prostate gland. The sequential method is very useful in circumstances when only a limited amount of tissue is available. Prostein, a 553 amino-acid protein is positive in most of the prostatic tumours while p63, a transcription factor belonging to the p53 family is a marker of urothelial differentiation. The authors have reported that none of the urothelial or prostatic cancers evaluated in the study has shown positivity for both markers. The profile characteristic for urothelial cancers (p63+/p501s-) showed 95.7% sensitivity, 100% specificity and 100% positive predictive value while the immunohistochemical profile suggesting a prostatic origin (p63-/p501s+) showed a 90.2% sensitivity, 100% specificity and also 100% positive predictive value. [50]. It seems that adenocarcinoma of prostate may expresses estrogen receptor a (ER-a) in stromal and basal cells while epithelial cells could express estrogen receptor b (ER-B) [51]. The authors have revealed that all cases of low and intermediate grade prostatic adenocarcinoma and 83% of high grade tumours express ER-b. Their study not only rises the issue of modulation of prostate adenocarcinoma by estrogens but also suggest that ER-b may represent a reliable marker which may be used in selected cases. In difficult cases of metastatic prostatic adenocarcinoma, FISH may be the most effective way of reaching an accurate diagnosis in many types of cancers including prostate adenocarcinoma [52]. Taylor et al. [53] have reported that TP53 and PTEN, which may be prostate cancer tumour suppressors are commonly altered in prostatic adenocarcinoma. The nuclear receptor coactivator NCOA2 may also be altered in some prostate cancers. FISH may also identify a narrow deletion on 3p14 which is associated with TMPRSS2-ERG fusion characteristic only for prostatic adenocarcinomas. This abnormality may be described in some cases of TMPRSS2-ERG in parallel with a PTEN loss [53].

## Conclusion

We present a rare pattern of prostatic adenocarcinoma metastatic to the skin. Immunohistochemistry for PSA and PSAP along with clinical and radiological examination and personal history were corroborated for the final diagnosis. For the histo/dermatopathologist it is important to recognize the plethora of various patterns displayed by cutaneous metastases of a prostatic adenocarcinoma. Last but not least, we should be aware that the PSA/PSAP might not be helpful in confirming the diagnosis if the skin lesion represents the extension of a poorly differentiated prostatic adenocarcinoma and other markers and/or methods need to be employed.

## Competing interests

The authors declare that they have no competing interests.

## Authors’ contributions

EBP: drafted the manuscript, provided histopathological material, took digital pictures, AGS: helped drafting the manuscript, provided clinical background and interpretation, RGW: provided advice on interpretation and took digital pictures, MS: provided histopathological research information, helped drafting the manuscript, KB: helped drafting the manuscript, provided basic research information and histopathological evaluation. All authors read and approved the final manuscript.
